# Neuron-Associated Transcriptional Enrichment Is Associated with Distinct Biological States and Predicts Poor Prognosis in Gastric Cancer

**DOI:** 10.1245/s10434-026-19666-2

**Published:** 2026-04-30

**Authors:** Masanori Oshi, Mashaal Dhir, John M. L. Ebos, Itaru Endo, Kazuaki Takabe

**Affiliations:** 1 Department of Gastroenterological Surgery, Yokohama City University Graduate School of Medicine, Yokohama, Japan; 2 Department of Surgical Oncology, Roswell Park Comprehensive Cancer Center, Buffalo, NY USA; 3 Department of Immunology, Roswell Park Comprehensive Cancer Center, Buffalo, NY USA; 4 Department of Surgery, University at Buffalo Jacobs School of Medicine and Biomedical Sciences, The State University of New York, Buffalo, NY USA; 5 Department of Breast Surgery and Oncology, Tokyo Medical University, Tokyo, Japan; 6 Division of Digestive and General Surgery, Niigata University Graduate School of Medical and Dental Sciences, Niigata, Japan; 7 Department of Breast Surgery, Fukushima Medical University School of Medicine, Fukushima, Japan

## Abstract

**Background:**

The peripheral nervous system can contribute to tumor development, progression, and metastasis; however, the clinical relevance of neuronal features within the tumor microenvironment in patients with gastric cancer (GC) remains to be fully elucidated.

**Patients and Methods:**

Bulk transcriptomic and clinical data from publicly available databases were used to evaluate an xCell score that quantified the relative enrichment of neuron-associated gene expression programs in GC tumors. This allowed stratification into neuron score-high and neuron score-low groups within each cohort, with the upper two-thirds defined as high. Associations between neuronal enrichment and tumor biology were evaluated through analyses of neurotransmitter receptor expression, gene set enrichment, tumor microenvironment composition, genomic features, and patient survival.

**Results:**

Neuron score-high GC exhibited characteristic enrichment of β-adrenergic, dopamine, and muscarinic acetylcholine receptor gene expression, consistent with enhanced neurochemical signaling potential. At the tumor cell level, the neuron score-high group was associated with activation of epithelial–mesenchymal transition (EMT) programs, whereas cell proliferation-related pathways were preferentially enriched in neuron score-low tumors. Neuron score-high tumors further displayed attenuated antitumor immune infiltration, increased stromal components, and elevated intratumor heterogeneity despite a lower mutation burden. Across both cohorts, neuron score-high tumors were associated with worse overall survival and remained independently associated with prognosis in multivariate analyses.

**Conclusions:**

Neuron score-high GC tumors were consistently associated with distinct patterns of neurotransmitter receptor gene expression, enhanced EMT programs with reduced cell proliferation, attenuated antitumor immune infiltration, increased intratumor heterogeneity, and worse prognosis. These observations identify the neuron score in bulk tumors as a potential indicator of GC biology.

**Supplementary Information:**

The online version contains supplementary material available at 10.1245/s10434-026-19666-2.

The tumor microenvironment (TME) plays a critical role in shaping cancer development, progression, and metastasis.^1,2^ In gastric cancer (GC), TME components such as immune cells, cancer-associated fibroblasts, endothelial cells, and extracellular matrix remodeling influence clinical outcomes and therapeutic responses.^3^ There is also growing evidence that peripheral nerves actively regulate components of the TME and impact tumor progression.^4–6^ To date, the relationship between the peripheral nervous system and GC has primarily been assessed using routine hematoxylin and eosin staining to determine perineural invasion (PNI), which is defined as cancer cell infiltration into the perineurium or neural fascicles.^7^ Although PNI is a well-established adverse prognostic factor in GC,^7–9^ it captures what is believed to be invasion of nerves by tumors and may not reflect the converse situation of nerve invasion of the TME. But a more extensive assessment of the reciprocal communication between peripheral nerves and tumors is needed. For instance, tumors recruit nerve fibers through neurotrophic signaling and induce neurogenesis,^10^ while neuron-derived signals promote cancer progression through mechanisms such as metabolic reprogramming^11^ and immunomodulation.^12,13^ These findings indicate that neural infiltration represents a biologically active component of the TME rather than merely a bystander phenomenon involving growth of tumors near local nerves. Indeed, nerves within the breast cancer TME have been shown to play a pivotal role in promoting tumor aggressiveness,^14,15^ and a high density of nerve fibers in the breast cancer TME has been correlated with poor survival outcomes.^16^ Currently, there is an unmet need in clinical practice for an objective measure to quantify nerve fibers in the TME, which largely relies on histological assessment. Indeed, Li and colleagues recently demonstrated that nerve fibers were detected in 85% of patients with breast cancer when entire tissue sections from each block were stained with two independent antibodies, whereas only 12–28% were detected when tissue microarrays were analyzed.^16^

In this regard, advancements in transcriptomic analytical methods may assist in further interrogating the relationship between neuron-associated programs within the GC TME. xCell is a gene signature-based computer algorithm that performs cell-type enrichment analysis from gene expression data for 64 immune, stromal, and stem cell types. Utilizing xCell in real-world patient cohorts linked with transcriptomes, our group has been able to quantify relative enrichment of specific cell-associated transcriptional programs and evaluate the clinical relevance of their presence in the TME, such as macrophages,^17^ dendritic cells,^18^ CD8^+^ cells,^19^ regulatory T cells,^20,21^ microvascular endothelial cells,^20^ and lymphatic endothelial cells.^22^ This approach has been particularly powerful for cell-associated programs that are extremely rare and would otherwise require entire tissue sections to be detected histologically, such as common myeloid progenitor cells.^23^

In this study, we sought to examine the clinical and biological significance of neuron-associated transcriptional enrichment in GC using a robust deconvolution framework and the xCell algorithm applied to publicly available GC datasets with linked clinical annotation, including The Cancer Genome Atlas (TCGA) and an independent Gene Expression Omnibus (GEO) cohort (GSE84437). Using this approach, we quantified relative enrichment of neuron-associated gene expression programs within bulk tumors and evaluated their associations with patient survival, neuron pathway enrichment, immune profiling, and genomic features.

## Patients and methods

### Cohorts and Transcriptomic data Processing

Transcriptomic and clinical data for GC were retrieved from TCGA stomach adenocarcinoma cohort (TCGA-STAD)^24^ and independent validation cohort GSE84437.^25^ For TCGA-STAD, gene-level expression values were derived from the RNA sequencing (RNA-seq) expression matrix and analyzed on the log-transformed scale (log_2_(TPM + 0.05)). For GSE84437, the publicly available microarray expression matrix was used as provided by the original study; when necessary, probe-level measurements were summarized to gene-level expression by selecting the probe with the highest average expression across samples. All analyses were conducted within each cohort independently to avoid cross-platform artifacts. Expression matrices were normalized and harmonized using standard procedures. Clinical annotations were harmonized using standard nomenclature and sample identifiers. Samples lacking the required transcriptomic profiles or the xCell neuron score were excluded from downstream analyses. Unless otherwise specified, all statistical analyses were performed in R (version 4.3.0).

### Inference of Neuronal Features Using xCell

Cell-type enrichment scores were inferred from bulk transcriptomes using xCel.^26^ Neuronal features were estimated using the xCell neuron score for each tumor sample, which represents the relative enrichment of a neuronal gene expression signature in bulk tumors rather than a direct quantification of histologically identifiable nerves or neural infiltration. The genes used for the neuron score are listed as Supplementary Table 1. Within each cohort, neuron scores exhibited a highly left-skewed distribution, with a substantial proportion of samples clustered near low values (Supplementary Fig. S1), indicating that biologically meaningful variation is concentrated toward the upper tail rather than varying linearly across the full range. To preserve biological contrast while maintaining adequate sample size, we adopted a distribution-based, quantile stratification strategy, classifying tumors in the bottom 33% of neuron scores distribution as neuron-low and remaining cases as neuron-high. This approach increases contrast while preserving adequate sample size for downstream survival and molecular analyses within each cohort. Mutation burden and intratumor heterogeneity metrics were obtained from previously published TCGA-derived datasets, specifically from the comprehensive immunogenomic analysis reported by Thorsson et al. In that study, intratumor heterogeneity was quantified on the basis of tumor subclonal architecture inferred from TCGA sequencing data. These precomputed metrics were integrated with neuron score stratification to evaluate the relationship between neuronal signature enrichment and tumor genomic features.

### Neurotransmitter Receptor Expression Analysis

To assess neurochemical signaling features associated with neuronal enrichment, the expression levels of selected neurotransmitter receptor genes, including β-adrenergic, dopamine, and muscarinic acetylcholine receptor families, were compared between neuron-high and neuron-low tumors within each cohort. Effect sizes were estimated using Hedges’ *g* with 95% confidence intervals. Results were visualized using forest plots to facilitate comparison of effect magnitude and direction across genes and cohorts. Statistical significance testing was performed separately and interpreted in conjunction with effect size estimates.

### Tumor Microenvironment Profiling and Functional Enrichment Analysis

To characterize TME differences associated with neuron enrichment, immune and stromal compartment features were evaluated using xCell-derived cell-type scores. Gene set enrichment analysis (GSEA) was performed using the Molecular Signatures Database (MSigDB) hallmark gene sets to identify biological pathways associated with neuron-high versus neuron-low tumors. For each cohort, genes were ranked according to the direction and magnitude of differential expression between neuron-high and neuron-low groups, and enrichment significance was assessed using standard permutation procedures implemented in established GSEA workflow.

### Statistical Analysis and Visualization

All statistical analyses were performed in R (version 4.3.0). Unless stated otherwise, all tests were two-sided. For comparisons involving two independent groups, statistical significance was assessed using the Wilcoxon rank-sum test (Mann–Whitney *U* test). For comparisons across more than two groups, the Kruskal–Wallis test was applied, followed by appropriate post hoc analyses when indicated. Multiple-testing correction was performed using the Benjamini–Hochberg method where applicable. Effect sizes are reported with 95% confidence intervals where relevant, particularly for analysis comparing neuron-high and neuron-low tumors. Overall survival was analyzed using Kaplan–Meier estimates with log-rank testing, and Cox proportional hazards regression was applied for univariate and multivariate survival analyses.

## Results

### Neuron Score-High GC Is Associated with High Expression of Neurotransmitter-Responsive Receptors

**Fig. 1:**
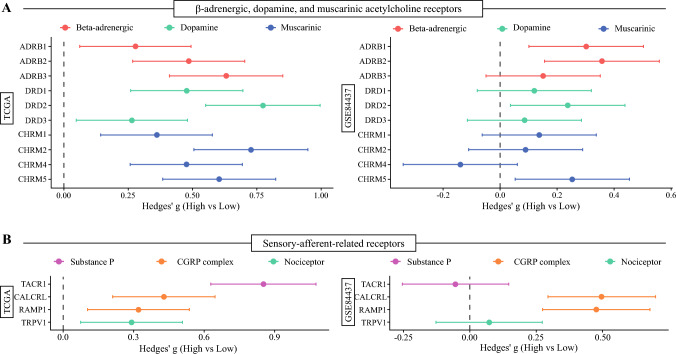
Association between neuron score and neurotransmitter receptor expression in gastric cancer; forest plots showing effect size estimates for differential expression of neurotransmitter receptor genes between neuron score-high and neuron score-low tumors within TCGA and GSE84437 cohorts; points indicate standardized effect sizes (Hedges’s *g*) for neuron score-high versus neuron score-low tumors, and horizontal bars represent 95% confidence intervals; genes are organized by receptor family, including β-adrenergic, dopamine, and muscarinic acetylcholine receptors, as well as sensory-afferent-related receptors (substance P receptor: TACR1, CGRP receptor complex; CALCRL/RAMP1, and nociceptor-associated receptor; TRPV1)

We decided to first compare the expression profiles of neurotransmitter receptor genes between tumors with high and low neuron score because communication between nerves and cancer cells within the TME is frequently mediated by neurotransmitter-dependent signaling pathways that influence tumor progression and metastasis. To this end, we quantified neuronal signature enrichment in each tumor using the xCell-derived neuron score based on a predefined neuronal gene set (Supplementary Table S1) and stratified samples accordingly, and then examined differential expression of neurotransmitter receptor genes between neuron score-high and neuron score-low tumors. In the TCGA cohort, tumors with high neuron scores consistently exhibited a marked higher expression of all examined neurotransmitter receptor genes, including β-adrenergic receptors (*ADRB1-3*), dopamine receptors (*DRD1-3*), muscarinic acetylcholine receptors (*CHRM1*, *CHRM2*, *CHRM4*, and *CHRM5*), and sensory-afferent-related signaling genes (*TACR1*, *CALCRL*, *RAMP1*, and *TRPV1*), compared with neuron score-low tumors (Fig. 1, left panel). The effect size estimation using Hedges’ *g* revealed moderate-to-large positive effects across these genes, with confidence intervals that did not overlap zero, indicating robust and biologically meaningful differences between the two groups. We next evaluated these associations in the GSE84437 cohort (Fig. 1, right panel). In this validation dataset, a more selective pattern emerged. Among sensory-afferent-related genes, components of the CGRP receptor complex, *CALCRL* and *RAMP1*, remained significantly upregulated in neuron score-high tumors, with consistent effect sizes and false discovery rate-adjusted significance across both cohorts. In contrast, *TACR1* (substance P receptor) and *TRPV1* exhibited cohort-dependent patterns, reaching statistical significance in TCGA but not in GSE84437. A similar trend was observed for several classical neurotransmitter receptors, for which the direction of effect was largely preserved but the magnitude of association was attenuated and accompanied by wider confidence intervals in the validation cohort. Taken together, these findings indicate that tumors enriched for neuronal features are associated with enhanced transcriptional responsiveness to multiple neurotransmitter modalities. Importantly, the consistent upregulation of the CGRP receptor axis across independent cohorts highlights sensory-afferent signaling as a reproducible neurochemical feature of neuron score-high GCs, while the cohort-specific behavior of other receptors suggests context-dependent engagement of distinct neuro–tumor communication pathways.

### Divergent EMT and Proliferative Programs in Neuron Score-High and Neuron Score-Low GCs

**Fig. 2:**
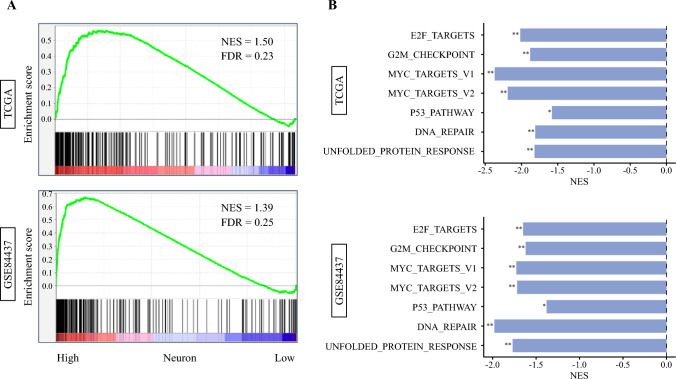
Transcriptional programs associated with neuron scores; **A** gene set enrichment analysis (GSEA) enrichment plots for epithelial–mesenchymal transition (EMT) hallmark gene set in both TCGA and GSE84437 cohorts; **B** bars indicate normalized enrichment scores (NES) for selected hallmark pathways, including E2F targets, G2M checkpoint, MYC target v1 and v2, TP53 pathway, DNA repair, and unfolded protein response, shown in a fixed order; asterisks denote pathways meeting the GSEA significance threshold (false discovery rate (FDR) < 0.25); double asterisks indicate pathways meeting a more stringent threshold (FDR < 0.05); NES and FDR were calculated using the GSEA framework

To elucidate broader biological programs associated with high neuron score, we next examined whether neuron scores were associated with distinct transcriptional programs related to important cancer biology. We focused on epithelial–mesenchymal transition (EMT) and cell cycle-associated pathways, which represent complementary axes of tumor phenotypic plasticity. Gene set enrichment analysis (GSEA) revealed a clear divergence in transcriptional programs associated with neuron score. The hallmark EMT gene set was consistently enriched in neuron score-high tumors across both the TCGA and GSE84437 cohorts (Fig. 2A). In contrast, neuron score-low tumors showed robust enrichment of cell cycle- and cell proliferation-related pathways, including E2F targets, G2M checkpoint, and MYC target gene sets, together with pathways related to DNA repair and unfolded protein response (Fig. 2B). All cell proliferation-related programs exhibited negative normalized enrichment scores, indicating preferential activation in neuron score-low tumors, whereas EMT was directionally enriched in neuron score-high tumors, with concordant patterns observed across both cohorts. Together, these findings delineate distinct transcriptional states associated with high neuron score, characterized by EMT-related programs in neuron score-high tumors and proliferative signaling in neuron score-low tumors.

### Immune Landscape Associated with High and Low Neuron Score

**Fig. 3:**
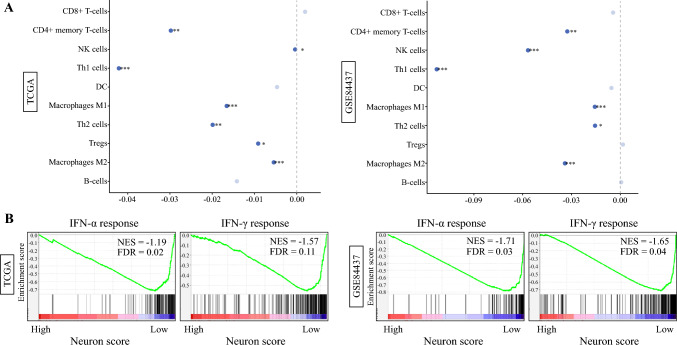
Association between neuron score and immune landscape in gastric cancer; **A** dot plots represent the median difference in cell-type enrichment scores, including CD8^+^ T cells, CD4^+^ memory T cells, NK cells, Th1 and Th2 cells, DC, macrophages M1 and M2, Tregs, and B cells, for each immune cell population in the TCGA and GSE84437 cohorts; asterisks denote adjusted significance levels (*false discovery rate (FDR) < 0.05; **FDR < 0.01; ***FDR < 0.001); **B** gene set enrichment analysis (GSEA) enrichment plots for immune-related hallmark pathways, including interferon (IFN)-α and IFN-γ response gene sets; normalized enrichment scores (NES) and FDR are indicated; pathways meeting the GSEA-recommended significance threshold (FDR < 0.25) were considered significant

To further characterize the TME associated with neuron score, we examined immune cell infiltration and immune-related transcriptional programs in neuron score-high and neuron score-low tumors. Using xCell-based deconvolution, neuron score-high tumors consistently exhibited reduced infiltration of multiple immune cell populations compared with neuron score-low tumors in both the TCGA and GSE84437 cohorts. In the TCGA cohort, significantly increased fractions of CD4^+^ memory T cells, Th1 cells, Th2 cells, regulatory T cells, natural killer (NK) cells, and both M1 and M2 macrophages were observed in neuron score-low tumors (Fig. 3A). Notably, macrophage subsets and Th1 cells showed particularly strong differences with robust statistical significance. A highly similar pattern was independently validated in the GSE84437 cohort, in which neuron score-low tumors likewise demonstrated significantly higher infiltration of CD4^+^ memory T cells, Th1 cells, Th2 cells, NK cells, and macrophage subsets, and Th1 cells showed particularly strong differences with robust statistical significance (Fig. 3A). Across both cohorts, the direction of effect was highly concordant, with most immune cell types displaying increased infiltration in neuron score-low tumors, despite differences in transcriptomic platforms and cohort composition. Consistent with these cellular-level findings, gene set enrichment analysis revealed attenuated immune-related transcriptional programs in neuron score-high tumors. Hallmark interferon (IFN)-α and IFN-γ response gene sets were significantly enriched in neuron score-low tumors in both cohorts, as indicated by negative normalized enrichment scores and false discovery rates below the GSE-recommended threshold (false discovery rate [FDR] < 0.25; Fig. 3B). These results collectively indicate that low neuron score GC is associated with an immune-infiltrated and interferon-responsive tumor microenvironment, reproducibly observed across independent gastric tumor cohorts.

### Tumor-Intrinsic and Stromal Features Associated with Neuron Score

**Fig. 4:**
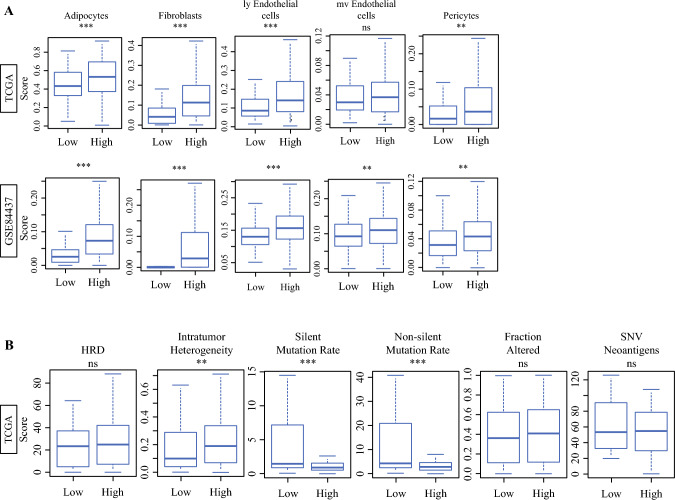
Stromal and genomic features associated with neuron score in gastric cancer; (**A**) box plots showing the infiltration fractions of stromal cell populations, including adipocytes, fibroblasts, lymphatic endothelial cells, microvascular endothelial cells, and pericytes, estimated using xCell in neuron score-high and neuron score-low tumors in both cohorts; (**B**) box plots comparing mutation-related genomic features, including homologous recombination deficiency (HRD), intratumor heterogeneity, silent and nonsilent mutation rate, fraction altered, and single-nucleotide variant (SNV) neoantigen load between neuron-high and neuron-low tumors in the TCGA cohort; box plots represent medians and interquartile ranges; asterisks denote adjusted significance levels (*false discovery rate (FDR) < 0.05; **FDR < 0.01; ***FDR < 0.001)

To further delineate nonimmune components of the TME associated with neuron score, we next examined stromal cell populations using xCell-based deconvolution. In contrast to the immune compartment, neuron score-high tumors exhibited consistently increased infiltration of multiple stromal cell types across both the TCGA and GSE84437 cohorts (Fig. 4A). In the TCGA cohort, neuron score-high tumors showed significantly higher enrichment scores for adipocytes, fibroblasts, lymphatic endothelial cells, and pericytes, with particularly pronounced differences observed for fibroblasts (Fig. 4A). A similar pattern was independently validated in the GSE84437 cohort, where neuron score-high tumors demonstrated significantly elevated levels of fibroblasts, lymphatic endothelial cells, microvascular endothelia cells, and pericytes. Although the magnitude of individual stromal components varied between cohorts, the direction of effect was highly concordant, with stromal infiltration consistently increased in neuron score-high tumors across platforms. To further explore genomic characteristics associated with neuron score, we analyzed mutation-related scores derived from publicly available TCGA datasets. Using mutation load-related metrics curated in the TCGA Pan-Cancer Atlas, neuron score-high tumors exhibited distinct genomic features compared with neuron score-low tumors (Fig. 4B). Specifically, neuron score-high tumors demonstrated significantly higher intratumor heterogeneity, whereas silent and nonsilent mutation rates were significantly increased in neuron score-low tumors. In contrast, no consistent differences were observed between neuron score-high and neuron score-low tumors with respect to homologous recombination deficiency, fraction of genome altered, or predicted single-nucleotide variant-derived neoantigen load. These findings indicate that neuron score is associated with specific patterns of genomic variation, characterized by increased intratumor heterogeneity and reduced overall mutational burden in neuron score-high tumors within the TCGA cohort.

### Neuron Score Identifies a Clinically Relevant Subgroup of Gastric Cancers

**Fig. 5:**
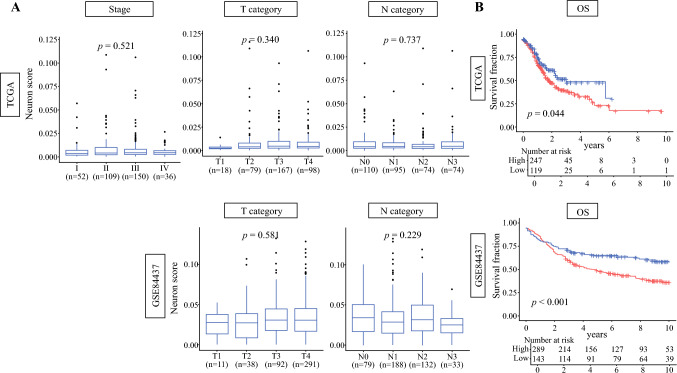
Clinical relevance of neuron score in gastric cancer; **A** box plots showing neuron scores stratified by pathological stage and T and N categories in the TCGA cohort, and by T and N categories in the GSE84437 cohort; **B** Kaplan–Meier curves of overall survival for neuron-high and neuron-low patients in both cohorts. Survival differences were assessed using the log-rank test

The data obtained from our analyses reported above suggested that there may be important differences in clinical outcome data based upon neuron scores. To assess this exciting possibility, we examined the relationship between neuron scores and clinicopathological parameters as well as patient survival. Neuron scores did not differ significantly across pathological stage or tumor extent in either cohort (Fig. 5A). In the TCGA cohort, neuron scores were comparable across AJCC stages as well as T and N categories. Similarly, in the GSE84437 cohort, no significant differences in neuron score were observed across T or N categories. However, despite the lack of association with pathological stage, neuron score was significantly associated with patient survival (Fig. 5B). Patients classified as neuron score-high exhibited significantly worse overall survival compared with neuron score-low patients in both cohorts (*p* = 0.044 in TCGA, and *p* < 0.001 in the GSE84437). In addition, in the TCGA cohort, neuron score-high tumors were significantly less likely to be MSI high, and ERBB2 mRNA expression was significantly higher in neuron score-low tumors (Supplementary Fig. S2A,B). We next examined the relationship between neuron score and TCGA molecular subtypes (CIN, EBV, GS, and MSI). The distribution of molecular subtypes differed significantly between neuron score-high and neuron score-low tumors (Supplementary Fig. S3A, *p* < 0.001), with neuron score-high tumors enriched for GS and CIN subtypes and neuron score-low tumors relatively enriched for MSI and EBV subtypes (Supplementary Fig. S3B). Together, these findings suggest that neuron score captures clinically relevant information not reflected by standard staging parameters.

### Neuron Score Is Independently Associated with Adverse Survival

Given that the Kaplan–Meier analyses demonstrated an association between neuron score and overall survival (OS) in Fig. 5, we finally assessed the prognostic value of neuron score using Cox proportional hazards regression analysis. Univariate and multivariate Cox regression analyses were performed separately in the TCGA and GSE84437 cohorts. Variables significantly associated with OS in univariate analyses were included in multivariate models. In both cohorts, high neuron score was significantly associated with worse OS in univariate analysis and remained independently associated with survival after multivariate adjustment (Table 1). In the TCGA cohort, patients with neuron score-high tumors exhibited an increased risk of death compared with those with neuron score-low tumors. This association was independently validated in the GSE84437 cohort, in which neuron score similarly remained significantly associated with OS.

**Table 1:** Univariate and multivariate Cox regression analyses for OS

TCGA (OS)		Univariate					Multivariate				
		HR	95% CI		*p*-Value		HR	95% CI		*p*-Value	
Age		1.02	1.01–	1.04	0.010	*	1.03	1.01–	1.04	0.005	*
Race	CA versus others	1.11	0.79–	1.58	0.543						
Gender	Male versus female	1.28	0.89–	1.82	0.181						
Grade	G3 versus G1/2	1.36	0.96–	1.93	0.083						
T	T3/4 versus T1/2	1.70	1.18–	2.58	0.013	*	1.44	0.92–	2.25	0.113	
N	N^+^ versus N^−^	1.93	1.27–	2.94	0.002	*	1.81	1.17–	2.81	0.008	*
Neurons	High versus low	1.46	1.01–	2.12	0.045	*	1.54	1.05–	2.26	0.026	*
GSE84437 (OS)		Univariate					Multivariate				
		HR	95% CI		*p*-Value		HR	95% CI		*p*-Value	
Age		1.02	1.01	–1.03	0.003	*	1.02	1.01–	1.03	< 0.001	*
Gender	Male versus female	1.26	0.93–	1.71	0.131						
T	T3/4 versus T1/2	3.77	1.93–	7.35	< 0.001	*	3.47	1.78–	6.78	< 0.001	*
N	N^+^ versus N^−^	2.05	1.36–	3.10	< 0.001	*	1.98	1.31–	2.99	0.001	*
Neurons	High versus low	1.73	1.26–	2.38	< 0.001	*	1.76	1.28–	2.43	< 0.001	*

*HR* hazard ratio, *CI* confidence interval

## Discussion

By integrating bulk transcriptomic analyses across two independent gastric cancer patient cohorts, we found that the neuron score of the tumor microenvironment can help to identify clinically and biologically distinct tumor states, revealing a previously underappreciated component of gastric tumor biology with potential relevance to disease progression. Specifically, we observed that neuron score-high tumors were not only associated with enrichment of neurotransmitter receptor gene expression but also enhanced epithelial–mesenchymal transition programs, attenuated antitumor immune infiltration, increased intratumor heterogeneity, and worse overall survival compared with tumors yielding a low neuron score. These data highlight a previously underappreciated component of gastric tumor biology with relevance to disease progression.

Accumulating evidence across multiple cancer types has highlighted a functional role for the nervous system in tumor development, progression, and metastatic dissemination.^6^ Experimental work further demonstrates that sympathetic denervation or pharmacologic β-adrenergic blockades can suppress tumor growth and metastasis, emphasizing the functional relevance of neuro–tumor interactions.^27–29^ In pancreatic cancer, sensory neuron infiltration has been shown to promote neural remodeling and support an invasive tumor phenotype.^30,31^ Beyond direct neural contact, neurochemical signaling pathways, including adrenergic and cholinergic axes, have been implicated in stress-mediated metastasis, stromal remodeling, and immune suppression.^32–34^

The present study demonstrated a systematic association between neuron scores in the tumor microenvironment and the expression of neurotransmitter receptor genes across large patient cohorts. Tumors classified as neuron score-high on the basis of xCell-defined neuron gene signatures exhibited increased expression of β-adrenergic, dopamine, and muscarinic acetylcholine receptor genes, together with enhanced expression of sensory-afferent-related receptors, most notably the calcitonin gene-related peptide (CGRP) receptor complex components CALCRL and RAMP1. This pattern was robust in the RNA-seq-based TCGA cohort and, despite reduced statistical power, largely preserved in direction in an independent microarray-based cohort. These findings suggest that neuron score-high tumors are transcriptionally poised to respond to diverse neurochemical cues, capturing a molecular footprint of neuro–tumor interactions that extends beyond histologically defined perineural invasion. While prior studies have focused on localized nerve–tumor contacts,^35,36^ our results indicate that neuronal influences may shape gene expression programs at a broader ecosystem level, interacting dynamically with immune and stromal compartments. Notably, CGRP signaling has emerged as a key modulator of vascular tone, stromal remodeling, and immune regulation in neoplastic tissues. We observed a consistent upregulation of the CGRP receptor complex (CALCRL/RAMP1) in neuron score-high gastric cancers across independent cohorts, therefore highlighting sensory-afferent CGRP signaling as a biologically relevant and reproducible axis of neuro–tumor communication. These results indicate that neuron score is not simply a surrogate for conventional measures of tumor burden or local invasion. In contrast, the cohort-dependent behavior of TACR1 and TRPV1 suggests that substance P- and nociceptor-related pathways may operate in a more context-specific manner, potentially shaped by tumor differentiation status, inflammatory milieu, or epithelial–mesenchymal transition programs. Together, these observations support a model in which neurochemical crosstalk in gastric cancer is hierarchically organized across sensory signaling axes, with CGRP-mediated pathways constituting a dominant and more reproducible feature of neuron score-high tumors.

EMT is a central program in tumor progression, frequently associated with metastatic competence, therapy resistance, and immune evasion. In this study, high neuron scores were consistently linked to an EMT-associated transcriptional state in gastric cancer, while tumors with lower neuron scores displayed a contrasting enrichment of cell proliferation programs. These observations align with emerging evidence that EMT signatures capture clinically meaningful tumor biology in gastric cancer.^37^ We previously reported that elevated EMT signatures are associated with adverse microenvironmental features, angiogenesis-related programs, and worse survival, and further reported an inverse relationship between EMT and cell proliferation-related signaling.^37^ Our findings extend this framework by placing EMT within a neuronal context: The EMT-enriched state preferentially mapped to neuron score-high tumors, whereas cell proliferation-related programs concentrated in neuron score-low tumors, with directional concordance across platforms. Together, these data suggest that neuron score may be coupled to a tumor cell state characterized by increased phenotypic plasticity rather than proliferative predominance. This interpretation is further supported by the observed enrichment of GS and CIN subtypes among neuron score-high tumors, as both subtypes have been linked to mesenchymal, stromal, or chromosomal instability-associated phenotypes in gastric cancer. In contrast, the relative enrichment of MSI and EBV subtypes in neuron score-low tumors is consistent with their more immune-active and less mesenchymal transcriptional profiles. The transcriptional association between neuron scores and EMT is biologically plausible in light of prior work implicating neurochemical signaling in cancer progression. β-adrenergic signaling has been broadly linked to tumor-promoting processes, including invasion and EMT-like transitions, and can engage canonical second-messenger pathways such as cAMP-PKA alongside context-dependent stress-response circuit.^34^ Consistent with this concept, the neuron score-high tumors identified in our analysis, characterized by coordinated upregulation of multiple neurotransmitter receptor genes, also exhibited preferential enrichment of EMT-associated transcriptional programs. At the same time, the divergent enrichment of EMT versus cell proliferation programs across neuron score-determined strata is compatible with a broader “state” model in which neuron score-high tumors preferentially occupy a plastic, invasion-associated phenotype, whereas neuron score-low tumors more strongly reflect a simpler cell proliferative biology.

A growing body of evidence indicates that the nervous system can modulate immune cell activity within the tumor microenvironment.^38–40^ Experimental studies have shown that adrenergic signaling can attenuate CD8^+^ T cell cytotoxicity, suppress NK cell function, and favor the accumulation of regulatory immune populations that promote tumor progression.^41,42^ In this context, our study provides a systematic transcriptomic perspective linking neuronal features to immune landscapes in gastric cancer. Specifically, neuron score-high tumors consistently exhibited reduced infiltration of multiple immune cell subsets and diminished IFN-related signaling across two independent cohorts. Although the present analyses do not establish causal relationships or delineate underlying mechanisms, the reproducibility and directional concordance of these findings suggest that neuron score is associated with a relatively immune-depleted tumor microenvironment. Importantly, this association was observed at the level of bulk transcriptional programs rather than focal nerve, tumor contacts, extending prior observations beyond histologically defined perineural invasion. Together, these results highlight the potential for neuron score to reveal a previously underappreciated axis of immune heterogeneity among gastric cancers with potential relevance to tumor–immune interactions and therapeutic responsiveness.

Intratumor heterogeneity represents a fundamental dimension of tumor complexity, reflecting the coexistence of diverse cellular states within a single lesion.^43–45^ In the present analysis, neuron score-high tumors were characterized by increased intratumor heterogeneity despite exhibiting lower rates of both silent and nonsilent mutations. This pattern indicates that genomic diversity in these tumors cannot be readily accounted for by mutation burden alone. Instead, it points to a decoupling between mutational load and heterogeneity in the context of high neuron score. Importantly, this elevated heterogeneity emerged in tumors that also displayed coordinated alterations in the tumor microenvironment, including reduced immune infiltration, expansion of stromal components, and enrichment of EMT-associated transcriptional programs. While the present study does not resolve the mechanisms underlying this convergence, the parallel emergence of these features across independent cohorts suggests that high neuron score marks a tumor context in which phenotypic diversification may be favored independently of ongoing mutagenesis. From this perspective, neuron score may delineate into a tumor ecosystem in which cellular diversity arises through adaptive states rather than genetic diversification, a hypothesis that warrants direct experimental interrogation.

In the present study, high neuron score of bulk tumors was consistently associated with worse overall survival across two independent gastric cancer cohorts. Importantly, this association remained significant in multivariate analyses incorporating established clinicopathological factors, indicating that neuron scores capture prognostic information beyond conventional clinical parameters. Notably, neuron score was not associated with tumor stage or local extent yet retained prognostic significance in survival analyses. This dissociation suggests that neuron score of the bulk tumor may reflect aspects of tumor biology that are not adequately represented by traditional staging systems. Rather than serving as a surrogate for tumor burden or anatomical progression, neuron score may index a distinct biological context linked to adverse clinical outcomes. The robustness of this association across cohorts profiled using different transcriptomic platforms further supports the generalizability of neuronal enrichment as a prognostic feature in gastric cancer. Although effect sizes varied between cohorts, the consistent directionality and independent significance argue against cohort-specific artifacts and underscore the biological relevance of neuron score within the tumor ecosystem. Moreover, the inverse associations between neuron score and MSI-high status, as well as ERBB2 expression, indicate that neuronal signature enrichment is related to, yet not simply interchangeable with, established molecular features, and instead reflects a distinct tumor state. Consistent with this, we also observed a significant association between neuron score and TCGA molecular subtypes, with neuron score-high tumors enriched for GS and CIN subtypes, whereas neuron score-low tumors were relatively enriched for MSI and EBV subtypes. This further supports the notion that neuronal signature enrichment is related to, but not reducible to, established molecular classification schemes. From a translational perspective, the xCell-derived neuron score should currently be regarded as a research-oriented metric based on bulk transcriptomic data, rather than a directly deployable clinical assay. While histopathological features such as nerve density or perineural invasion have been extensively studied, the present work addresses a different layer of tumor biology by capturing a transcriptional state reflecting neuronal signaling responsiveness within the tumor ecosystem. Rather than quantifying neural structures per se, our approach aims to identify a biologically and clinically relevant tumor state associated with adverse outcomes. In this regard, the neuron score provides a conceptual framework for molecular risk stratification and for generating hypotheses regarding neuro–tumor interaction-targeted therapeutic strategies in the future. Future studies integrating transcriptomic profiling with spatial and pathology-based assessment will be required to determine whether practical surrogate assays can be developed for clinical application.

Despite these interesting findings, several limitations warrant consideration. The neuron score used here is inferred from bulk transcriptomic data, and we cannot directly confirm that the score reflects histologically identifiable neurons or nerve density within the tumor microenvironment. Moreover, peripheral nerve fibers lack neuronal cell bodies, resulting in limited transcript abundance, and several genes included in the xCell neuron signature e.g., NEUROD2, NEUROG2, GAD2, ELAVL3, and STMN2 are classically associated with the central nervous system. As such, it is possible that the neuron score captures transcriptional programs from non-neuronal cell types. Future studies aimed at defining the cellular sources of neuron-associated transcriptional signals within tumors will therefore be critical. In addition, the observational nature of this study precludes causal inference regarding the functional role of neurotransmitter signaling in tumor progression. Future studies integrating spatial transcriptomics, functional perturbation, and longitudinal clinical data will be required to delineate the precise mechanisms underlying neuro–tumor communication. Another important limitation is the lack of uniformly annotated treatment response data in the publicly available transcriptomic cohorts analyzed here, which precludes direct evaluation of the relationship between neuron score and therapeutic response. Addressing this question will require future studies using clinically annotated cohorts or prospective clinical trial datasets with detailed treatment and outcome information.

## Conclusions

Our findings reveal that gastric cancers with high neuron score were consistently associated with distinct patterns of neurotransmitter receptor gene expression, enhanced epithelial–mesenchymal transition programs, attenuated antitumor immune infiltration, increased intratumor heterogeneity, and worse prognosis. These observations identify neuron score in bulk tumors as a previously underappreciated marker of gastric cancer biology. While the present study does not establish causality, it provides a framework for future investigations into neuro–tumor interactions and suggests potential avenues for patient stratification and therapeutic exploration.

## Supplementary Information

Below is the link to the electronic supplementary material.

Supplementary file1 (DOCX 531 KB)

## Data Availability

The Cancer Genome Atlas (TCGA) and GSE84437 are all publicly available without any restrictions via cBioportal or Gene Expression Omnibus (GEO).
