# Supplementary material for: Neuron-Associated Transcriptional Enrichment Is Associated with Distinct Biological States and Predicts Poor Prognosis in Gastric Cancer
Source: Ann Surg Oncol. 2026 Apr 30;33(9):8122–33. doi: 10.1245/s10434-026-19666-2 (PMC13452835; doi:10.1245/s10434-026-19666-2)
Supplement: Supplementary file 1 [file 10434_2026_19666_MOESM1_ESM.docx]

Supplemental data

Table 1. Gene set used for calculation of the neuron score.

EPHA3, GNG3, INSM1, KCNQ2, NEUROD2, PCDH, ACTL6B, CAMKV, STMN4, ABCA3, ACVR2B, ADRA2A, CACNA1B, CACNB3, CHRNB2, CPE, CRABP1, CRMP1, NCAN, CTNNA1, EFNB3, CELSR3, ELAVL3, FOXG1, GAD2, GAP43, GEM, GRIK3, GRM2, ID1, IREB2, KCNN1, KCNQ3, KLC1, MEIS2, MLF1, MLLT3, MN1, CD200, MVD, NNAT, NPY, NPTX1, NTRK3, PLXNA2, POU3F1, POU3F2, POU3F3, PKIA, MAPK8, PTPRZ1, PTX3, RPE65, SCN3A, CXCL12, SFRP4, SH3GL2	SH3GL3, SOX4, SOX11, SPAST, ZNF354A, THRA, ZFP37, ZNF711, ZNF14, ZNF43, ZNF195, ZNF223, TUBA1A, ST8SIA4, PDHX, ST8SIA2, HIST1H3D, DCHS1, STX16, B3GALT2, CDK5R1, BSN, HAP1, CYTH2, LHX2, IPO13, NUP93, TSPAN2, CFDP1, SEMA6C, ZNF211, IFI44, DPYSL4, SCGN, PNMA2, ZBTB6, MLLT11, TMSB15A, STMN2, C14orf1, ZFP30, ZNF510, WDR47, KIF21B, ARC, KIAA1107, AGTPBP1, CUX2, SEZ6L, SULT4A1, SETBP1, LRRTM, RANBP6, CACNG5, CACNG4, PDLIM3, PCSK1N, KCNMB, SCG3, HUNK, PODXL2, PARD6A, ZNF117, GPR173, FAM105A, LRRN3, FNBP1L, ENOX1, AGPAT5, AP1AR, ZNF821, ANKRD10, ZNF415, BEX1, PCDHB11, ZNF253, C21orf62, KCNK12, ZNF529, PTBP2, ZBED5, NEUROG2, NEUROD6, REEP1, RASL11B, GDAP1L1, NKAIN1, RNF219, ZMAT4, VASH2, ZNF669, PGAP1, ZNF606, ZNF614, ZNF43, ZSCAN16, ZNF34, ZNF484, YIPF4, MUM1, C16orf45, ZNF682, C1orf216, EMID1, PAQR3, ZNF675, TET3, KCTD13, ZNF493, ALDOC, ATP1A3, ATP1B1, ATP2B2, ATP6V1G2, CA8, CACNB4, CALB1, CAMK2B, CDH18, COX7A1, COX7B, DGKB, DEFB1, DLG2, DPP6, FABP6, FGF9, FGF12, GABBR1, GABRA1, GABRB2, GABRG2, GAD1, GNAO1, GPM6A, GRIA2, GRID2, GRIK1, GRM1, GRM7, ID2, ITPR1, KCNC1, KIF5C, LPL, MT3, NDUFA5, NEFM, NEFH, NELL1, NEFL, NPPC, OMG, PCDH9, PCP4, SERPINI1, PRKCG, PTPRR, PVALB, RGS16, RORA, RTN1, SLC6A1, SNAP25, SNCG, SYP, TAC1, SEC62, TSPAN7, TRPC3, ZNF208, AP3B2, SPARCL1, CACNA1G, INA, NRXN1, ELMO1, SNAP91, C1orf61, CNKSR2, FAIM2, ARHGAP26, FSTL4, ACSL6, SMPX, SLC24A2, DDX25, SPOCK3, ST8SIA3, GPRC5B, GNG13, TM6SF1, FXYD7, IL20RA, SUSD4, PRMT8, DNAJC12, ANKS1B, PLXDC1, SLC12A5, KLHL1, GPR63, TRIM9, TCEAL2, SHISA6, ACYP2, AGTR2, ATP1B2, ATP2A3, CA7, CACNA1A, CACNB2, CHGB, DAB1, DACH1, DGKG, DYNC1I1, FABP3, FGF14, GABRB3, GNG4, GRIA3, HSBP1, HTR5A, KCNA2, KCNK1, KIF5A, KPNA5, NAP1L2, NCAM1, NDUFA3, NDUFA4, NELL2, NRCAM, PDE9A, PEG3, PPP3CA, MAPK10, RAB3A, RGS7, RYR2, SCN1A, SCN2A, SLC1A6, SLC8A1, ABCC8, VAMP2, KCNAB1, PIP5K1B, PPFIA2, SYNJ1, CACNA2D2, CPNE6, RAB33A, NRXN3, AKAP7, GABBR2, GPRASP1, JAKMIP2, SAP18, CORO2B, ATP5L, KIF3A, HHLA3, MGAT4A, GABARAPL2, KIFAP3, SV2C, MYT1L, DNM3, GALNT8, CLUL1, PCDH17, GOLIM4, PCLO, NDUFAF4, NME7, PIGP, BCL11A, FAM134B, LRRC49, CISD1, TTYH1, NDRG4, FAM184A, ZNF385D, MAP9, CEP76, HOPX, IQCK, RALYL, THSD7A, LPCAT4, FAM21A, ANK2, CHN1, FABP7, GPM6B, LHX1, NOVA1, PTPRN, SORL1, NME5, SPOCK2, RCAN2, KLK8, SOSTDC1, SNX10.

Supplemental figure:

**Figure S1. Distribution of neuron scores in the TCGA-STAD and GSE84437 cohorts.** Histogram shows the distribution of xCell-derived neuron scores across tumors in the TCGA-STAD (left) and GSE84437 (right) cohorts.

**Figure S2. Association between neuron score and established molecular biomarkers in TCGA cohort. (A)** Proportion of MSI-high tumors in neuron score-low and -high groups. Group differences were evaluated using Fisher’s exact test. **(B)** *ERBB2* mRNA expression levels in neuron score-low and -high tumors. Group differences were assessed using the Wilcoxon rank-sum test.

**Figure S3. Association between neuron score and TCGA molecular subtypes in TCGA-STAD. (A)** Stacked bar plot showing the proportional distribution of TCGA molecular subtypes (CIN, EBV, GS, and MSI) in neuron score-low and -high tumors. Group differences were evaluated using the chi-squared test. **(B)** Boxplots showing the distribution of neuron scores across TCGA molecular subtypes.
